# Bentonite- and Palygorskite-Based Gels for Topical Drug Delivery Applications

**DOI:** 10.3390/pharmaceutics15041253

**Published:** 2023-04-16

**Authors:** Marco Ruggeri, Rita Sánchez-Espejo, Luca Casula, Giuseppina Sandri, Luana Perioli, Maria Cristina Cardia, Francesco Lai, César Viseras

**Affiliations:** 1 Department of Drug Sciences, University of Pavia, Viale Taramelli 12, 27100 Pavia, Italy; marco.ruggeri@unipv.it (M.R.); giuseppina.sandri@unipv.it (G.S.); 2 Department of Pharmacy and Pharmaceutical Technology, University of Granada, 18071 Granada, Spain; ritamsanchez@ugr.es; 3 Department of Life and Environmental Sciences, Unit of Drug Sciences, University of Cagliari, 09124 Cagliari, Italy; luca.casula@unica.it (L.C.); cardiamr@unica.it (M.C.C.); frlai@unica.it (F.L.); 4 Department of Pharmaceutical Sciences, University of Perugia, Via del Liceo 1, 06123 Perugia, Italy; luana.perioli@unipg.it

**Keywords:** diclofenac acid, nanocrystals, clays, dermal delivery

## Abstract

Bentonite or palygorskite-based hydrogels have recently been suggested as a strategy to increase bioavailability and control the retention and release of therapeutic candidates. In this work, clay-based hydrogels loaded with diclofenac acid nanocrystals have been successfully designed and developed. The aim was to improve diclofenac solubility, its dissolution rate and to enhance its local bioavailability after topical application. For this purpose, diclofenac acid nanocrystals were prepared by wet media milling technology and then loaded into inorganic hydrogels based on bentonite and/or palygorskite. Diclofenac acid nanocrystals were characterized by morphology, size, and zeta potential. Moreover, rheological behavior, morphology, solid state, release studies, and in vitro skin penetration/permeation of diclofenac acid nanocrystals-loaded hydrogels were performed. The hydrogels were characterized by a crystalline structure, and demonstrated that the inclusion of diclofenac in clay-based hydrogels resulted in an increased thermal stability. The presence of both palygorskite and bentonite reduced nanocrystal mobility, and consequently its release and penetration into the skin. On the other hand, bentonite- or palygorskite-based hydrogels revealed great potential as an alternative strategy to enhance topical bioavailability of DCF nanocrystals, enhancing their penetration to the deeper skin layers.

## 1. Introduction

In the pharmaceutical field, the use of clays extends back into pre-history as both excipients and active ingredients. Archaeological evidence revealed that Homo erectus and Homo neanderthalensis used clay for wounds and skin irritations, and in the ancient world, clay-containing earths were frequently used to treat digestive and intestinal disorders [1]. To date, clays are applied via the oral route as antacids, gastrointestinal protectors and anti-diarrheics, and topically in the form of creams, emulsions, and gels, due to their capability to increase stability and the viscosity of suspensions [2].

Bentonite is a natural clay mineral composed mainly of montmorillonite (hydrated aluminum silicate). The crystalline structure is based on two layers of silica tetrahedron located between an octahedron aluminum core layer, constituting a lamella of about 1 nm. Inside the galleries there are cations generally represented by Na^+^ ions which counterbalance the negative charge of the surface of the lamellae, originating from the replacement of Si^4+^ with Al^3+^ in the tetrahedral layers, or Al^3+^ with magnesium Mg^2+^, Fe^2+^ or Ca^2+^ in the octahedral layers [3].

Palygorskite is a kind of hydrous magnesium–aluminosilicate clay mineral. It has a chain lamellar structure and its crystal structure is rod-like, fiber-like or fiber aggregate. Al^3+^ replaces Si^4+^ in the tetrahedron, creating negative charges on the surface [4]. Under the influence of electrostatic forces, both bentonite and palygorskite produce in water a random 3D network structure of inorganic gels [5,6]. These hydrogels can be used as adsorbents, thickeners, suspension agents, and stabilizers. They are widely used in the petrochemical industry, daily chemical industry (carrier for toothpaste and cosmetics), fine chemical industry (carrier for catalysts), pharmaceutical industry, pesticide, oil recovery, metallurgy, and building materials [7]. Additionally, bentonite- or palygorskite-based hydrogels have recently been suggested as a strategy to increase bioavailability and control the retention and release of therapeutic candidates [8,9].

Diclofenac acid (DCF), a non-steroid anti-inflammatory agent, exerts its therapeutic effects via inhibition of the cyclooxygenase I (COX 1) and cyclooxygenase II (COX II). Diclofenac acid is a drug that relieves pain, acts as an antifebrile and an anti-inflammatory, and is frequently used to treat rheumatoid arthritis, osteoarthritis, spastic spondylitis, acute gout, inflammation, and postoperative gout. Due to various drawbacks associated with oral administration such as peptide ulcers, DCF is most frequently topically administered in long-term therapy, such as in local muscle inflammation. Because of its relatively weak aqueous solubility, DCF is mainly available in sodium, potassium, and alkyl-hydroxyl amine salt form in commercial topical products [10]. To date, there are few commercial products based on DCF. Although its lipophilic nature can promote skin permeation, the poor aqueous solubility leads to a slow release which hinders penetration and permeation into and through the skin.

Particle size reduction to the nanosized range through nanocrystalline technology could resolve or improve DCF solubility, changing its physico-chemical properties and improving drug bioavailability [11,12]. Nanocrystals are usually manufactured as aqueous nanosuspensions and stabilized by ionic or non-ionic surfactants, polymers or a mixture of both. In order to enhance its permeation and absorption through the skin, aqueous DCF nanosuspensions have recently been administered in combination with medical devices, such as microneedle rollers [13] and needle-free jet injectors [14].

Nevertheless, another approach consists of using hydrogels as vehicles for nanocrystal suspensions [15]. In this work, clay-based hydrogels loaded with diclofenac acid nanocrystals have been designed and developed to improve diclofenac solubility, its dissolution rate and to enhance its local bioavailability after topical application. For this purpose, DCF nanocrystal suspensions were prepared by wet media milling technology and then loaded to inorganic hydrogels based on bentonite and/or palygorskite. DCF nanocrystals were characterized by morphology (SEM), size, and zeta potential. Moreover, rheological behavior, morphology (SEM), solid state, release studies, and skin penetration/permeation of DCF-loaded hydrogels were performed.

## 2. Materials and Methods

### 2.1. Materials

Diclofenac sodium salt powder and Poloxamer 188 (P188) were supplied respectively by Galeno (Comeana, Italy) and Sigma Aldrich (Milan, Italy). Bentonite and palygorskite (Pharmasorb^®^ Colloidal) were purchased from Sigma Aldrich (Madrid, Spain) and from Basf (Ludwigshafen, Germany), respectively. All HPLC solvents and reagents used were obtained from Sigma Aldrich (Madrid, Spain).

### 2.2. Methods

#### 2.2.1. Synthesis of DCF and Preparation of DCF Nanosuspension

DCF was prepared from a sodium salt solution following a published procedure by Pireddu et al., using diluted hydrochloric acid [12]. The obtained solid white precipitate of DCF was filtered, carefully washed with bidistilled water and dried. To prepare the nanosuspension, the drug was firstly added to an aqueous solution of P188 and homogenized by means of a rotor-stator homogenizer (Ultra Turrax T25 basic—IKA, Staufen, Germany) for 5 min at 6500 rpm. Secondly, the obtained suspension was transferred in conical tubes, and 0.1–0.2 mm yttrium-stabilized zirconia–silica beads were added to perform the milling. The tubes were then oscillated at 3000 rpm for 60 min by means of a bead-milling cell disruptor device (Disruptor Genie^®^, Scientific Industries, Bohemia, NY, USA). Finally, sieving was carried out to separate the milling beads from the formulation. The resulting nanosuspension had a concentration of 10 mg/mL DCF and 5 mg/mL P188 (Table 1).

#### 2.2.2. DCF Nanosuspension Characterization

Zetasizer nano (Malvern Instrument, Worcestershire, UK) was used to estimate the average diameter and polydispersity index (PDI, as a measure of the size distribution width) of the DCF nanosuspension by Dynamic Light Scattering (DLS). Samples were backscattered by a helium–neon laser (633 nm) at an angle of 173° and a constant temperature of 25 °C. Using the same instrument, the zeta potential of nanocrystals was evaluated by electrophoretic light scattering after dilution of the nanosuspension with distilled water (1:100).

Nanocrystals morphology was assessed by scanning electron microscopy. The samples were placed on metal stubs using a carbon double-sided adhesive tape sputtered with gold, and the images were captured using a S-4100 Hitachi (Oberkochen, Germany) at 10 kV.

#### 2.2.3. Preparation of DCF Nanosuspension-Loaded Hydrogels

Bentonite (30% *w*/*w*), palygorskite (30% *w*/*w*), or bentonite/palygorskite (15% *w*/*w* for each clay) were dispersed in distilled water and homogenized using an Ultra-Turrax T25 agitator (IKA, Staufen, Germany) for 10 min at 24000 rpm [15].

Regarding the hydrogels containing DCF nanocrystals, DCF nanocrystals were combined in a weight-to-weight ratio of 1:1, with the hydrogels previously prepared to obtain a final DCF concentration of 0.5% *w*/*w*. As previously described, the mixture was homogenized until DCF nanocrystals-loaded hydrogels (B-DCF, P-DCF, or B/P-DCF) were produced.

#### 2.2.4. Rheological Analysis

A Haake viscometer RotoVisco 1 (Thermo Scientific, Karlsruhe, Germany) equipped with a plate/plate combination (Ø 20 mm serrated PP20S sensor system) was used to carry out the rheological analysis of the hydrogels. A Peltier temperature controller was used to set the temperature at 32 °C. The analyses were performed in the shear rate range from 10 to 500 1/s.

#### 2.2.5. Solid State Characterization

Freeze-dried hydrogels were prepared by means of CoolSafe Touch 100-9 (LaboGene, Aarhus, Denmark) and used for solid state analysis.

The morphology of the hydrogels was investigated using a Hitachi S-510 microscope (Hitachi Scientific Instruments Ltd., Tokyo, Japan) at 25 kV in low vacuum mode (Thermo Scientific Quattro ESEM) equipped with an EDX analyzer.

X-ray powder diffraction (XRPD) analysis was performed using a diffractometer (X’Pert Pro model, Malven Panalytical, Madrid, Spain). The diffractogram patterns were recorded using CuKα radiation, operating at 45 kV and 40 mA, in the range 4–60° 2θ.

Fourier transform infrared (FT-IR) analysis was carried out using a FTIR spectrophotometer (JASCO 6200, Pfungstadt, Germany) equipped with a Ge ATR. All analyses were performed from 400 to 4000 cm^−1^ with a resolution of 0.25 cm^−1^, and the spectra was processed with Spectra Manager v2 software.

Thermal analysis (thermogravimetric and differential scanning calorimetry analysis) was assessed using TGA/DSC1 equipment (Mettler-Toledo, Madrid, Spain) [15].

#### 2.2.6. In Vitro Release Study of DCF Nanosuspension-Loaded Hydrogels

The in vitro release study was performed by means of Franz diffusion cells (PermeGear, Riegelsville, PA, USA) with an effective diffusion area of 0.9 cm^2^. The receptor chamber was filled with phosphate buffer pH 7.4 (PBS), continuously stirred at 50 rpm with a magnetic bar and separated from the donor chamber by an HV membrane (0.45 μm, Millipore, Ireland). The release studies were performed at 37 °C to maintain a membrane surface temperature at 32 °C [16,17,18]. An amount of 100 mg from each hydrogel was placed onto the membrane. At prefixed time intervals, 1 mL of dissolution medium was collected using a Hamilton^®^ syringe, and the sample volume was replaced in the receptor chamber to guarantee a constant volume as well as sink conditions.

The samples were analyzed using a Perkin Elmer HPLC series 200 system (Waltham, MA, USA). A Zorbax SB-C18 column (4.6 × 150 mm; 5 µm) was used as stationary phase, acetonitrile 70% (*v*/*v*) as mobile phase, the wavelength at 280 nm, the injection volume was 10 μL, the flow rate was 0.7 mL/min, and column temperature of the oven was set at room temperature.

#### 2.2.7. In Vitro Skin Penetration/Permeation

In vitro permeation tests were carried out using skin ear obtained from pigs donated by a nearby slaughterhouse (Granada, Spain). The subcutaneous fat was carefully removed and stored at −20 °C [15]. The skin was mounted between the donor and receptor camber of a Franz-type diffusion cell with an effective diffusion area of 0.9 cm^2^, with the stratum corneum facing the donor phase. The receptor phase was filled with PBS, continuously stirred by a magnetic bar and thermostated at 37 °C to reach skin temperature at 32 °C [16,17,18].

Each hydrogel (100 mg) was applied onto the stratum corneum of the skin. After 24 h, the skin surface was cleaned, and each of the three tissues (stratum corneum, epidermis, and dermis) was separated as described by Ruggeri et al. [15] and placed in methanol before HPLC analysis.

#### 2.2.8. Statistical Analysis

The data were subjected to statistical analysis by means of Astatsa statistical calculator. Analysis of variance (ANOVA) followed by Scheffé test were used to compare the experimental values.

## 3. Results and Discussion

### 3.1. DCF Nanosuspension Characterization

With the aim of obtaining a homogeneous water-based nanosuspension, drug nanocrystals were prepared by a wet ball media milling technique. Following previous studies, DCF concentration was fixed at 1% (*w*/*w*), and Poloxamer 188 at 0.5% (*w*/*w*) was used as a non-toxic stabilizer [12,19].

As reported in Table 1, DLS analysis on the day of preparation revealed an average nanocrystals diameter of approximately 288 nm with a polydispersity index (PDI) of 0.25, highlighting a narrow size distribution. With regards to the zeta potential determination, the sample showed a relatively negative value (−26.8 mV), indicating a promising stability of the nanosuspension. This was also confirmed by a previous stability study of the formulation over 90 days of storage [12]. Moreover, the drug is well known for having a long-term chemical stability in aqueous solutions and in the form of nanosuspensions [20,21].

**Figure 1:**
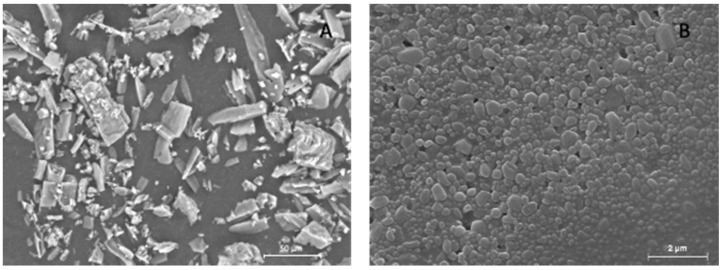
Scanning electron microscopy images of DCF raw material (**A**) and DCF nanocrystals (**B**).

To further investigate the morphology of the crystals and the effect of the nanosizing procedure, DCF was analyzed as bulk powder and as nanosuspension using SEM. Since DCF raw material/bulk powder appears as irregular thin and elongated crystals (Figure 1A), the effect of the high shear forces generated during the milling on the final product is evident. In fact, the nanocrystals present a regular and rounded shape with a homogeneous morphology and distribution (Figure 1B). Although nanocrystals evidenced a slight tendency to aggregate, this could be related to the method of preparing the samples for SEM analysis. In fact, a drop of formulation was put on the stub and left to dry before gold sputtering. The resultant evaporation of water may lead to an agglomeration of the nanocrystals, which is merely an operative artifact, as the PDI value obtained by the DLS analysis shows a narrow size distribution.

### 3.2. Rheological Properties

**Figure 2:**
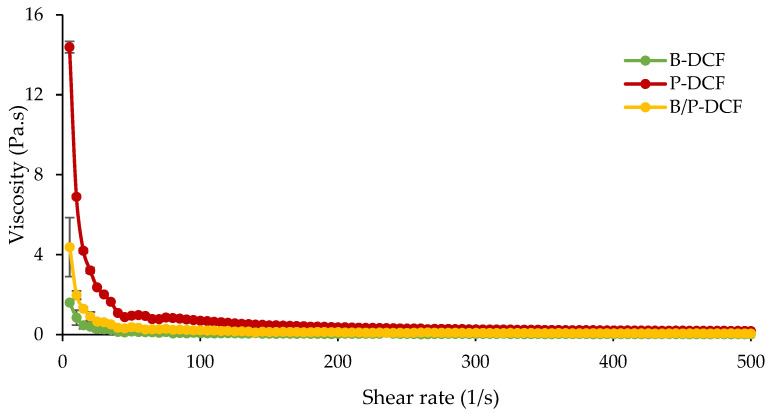
Viscosity behavior of B-DCF, P-DCF, and B/P-DCF hydrogels in the range of 10–500 1/s at 32 °C (mean values ± s.d.; n = 3).

The viscosity behavior of the hydrogels B-DCF, P-DCF, and B/P-DCF are shown in Figure 2. B-DCF hydrogel revealed a pseudoplastic non-Newtonian flow behavior, with a viscosity reduction when the shear rate increased. P-DCF hydrogel also showed pseudoplastic properties, and its viscosity values at low shear rates were seven times higher than those of B-DCF. This behavior could be related to the elongated thin particles of palygorskite causing high physical viscosity when added to water. Moreover, B/P-DCF hydrogel revealed a similar behavior, although its viscosity values were around two-fold higher than B-DCF hydrogel, and four-fold lower than those of P-DCF hydrogel. The pseudoplastic behavior of these hydrogels could be related to complex particle–particle association and the formation of a network between clay particles [22]. According to Luckham and Rossi [23], the reduction in viscosity as the shear rate increased could be related to the gradual destruction of networking structures, the reduction of the microaggregate size, and the orientation of the individual platelets towards the direction of flow [24]. After complete rupture of the particle–particle linkage, a constant viscosity at a higher shear rate is reached.

The addition of bentonite to palygorskite drastically modified the hydrogel rheological properties. The viscosity differences between P-DCF and B/P-DCF could be related to different factors. The individual rods or small crystal bundles in the hydrogel of pure palygorskite are oriented randomly and linked together to form clusters. These clusters contain a large number of water molecules, increasing the effective solid volume fraction and viscosity of the suspension. On the other hand, the decrease in viscosity for B/P-DCF may be attributed to an increase in interparticle attractive forces and a loss of interparticle repulsive forces between heterogenous particles, causing the hydrogel network to collapse. Similar results have been reported by Neaman et al., which demonstrated that larger additions of 10–20 wt% of montmorillonite to palygorskite caused a decrease in viscosity [25].

### 3.3. Solid State Characterization

**Figure 3:**
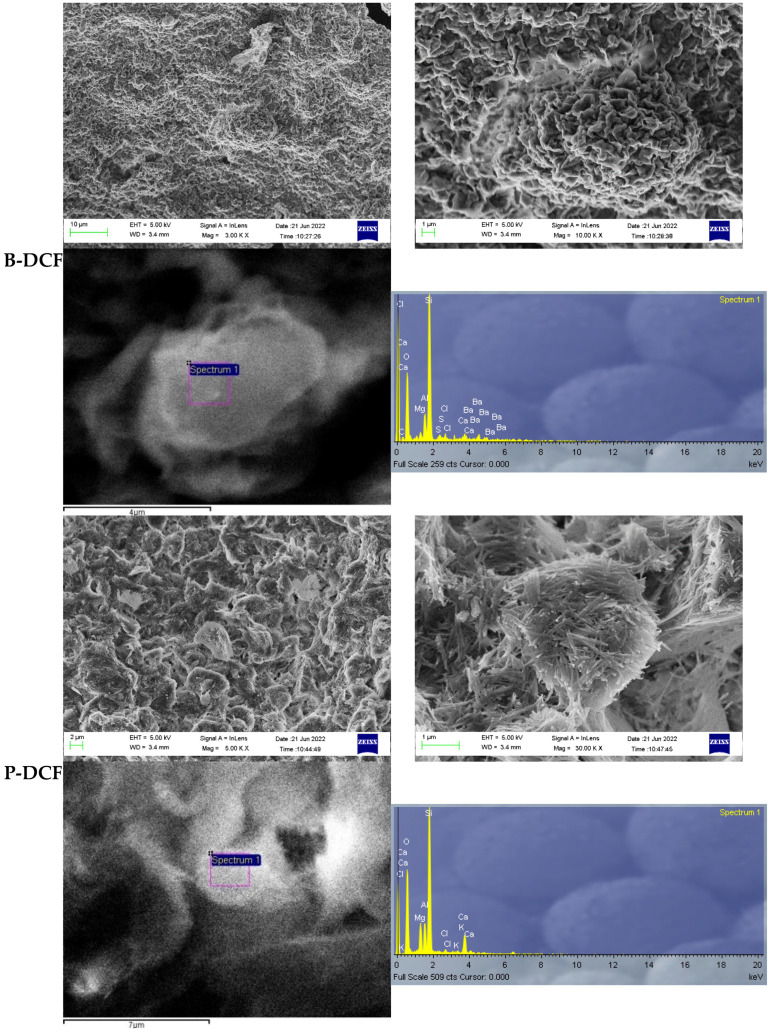

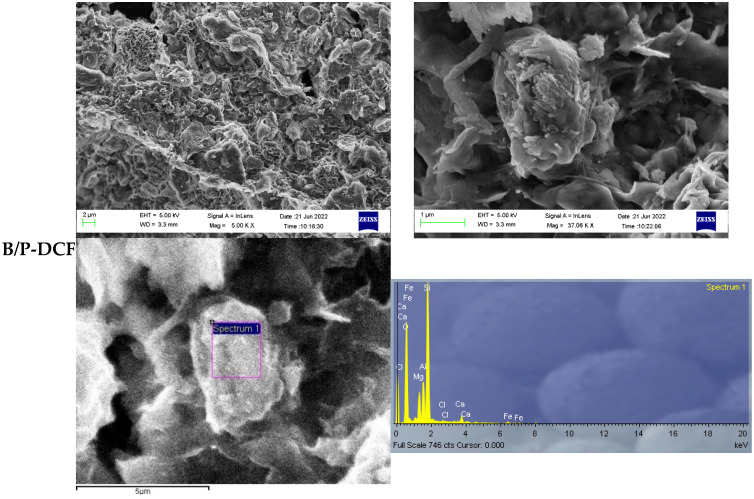
SEM microphotographs and EDS analysis of freeze-dried B-DCF, P-DCF, and B/P-DCF gels.

The B-DCF, P-DCF, and B/P-DCF gels’ SEM microphotographs are shown in Figure 3. The presence of different clays had a significant impact on the morphologies. The porous structure of B-DCF hydrogels, which had a flake-like structure, was due to the sublimation of water during the freeze-drying process. On the other hand, P-DCF gel displayed palygorskite-typical features, with several individual elongated needles of different sizes and bundles of elongate and lath-like particles. The morphological analysis of B/P-DCF gel revealed that the single crystals of palygorskite are inclined to aggregate into the lamellar curly surface of bentonite.

An energy-dispersive X-ray analyzer (EDX) was used to determine the chemical composition of DCF-loaded gels. The EDX spectra suggested Si and Ca as dominant in the hydrogels. Moreover, Cl found in small quantities confirms the presence of DCF.

**Figure 4:**
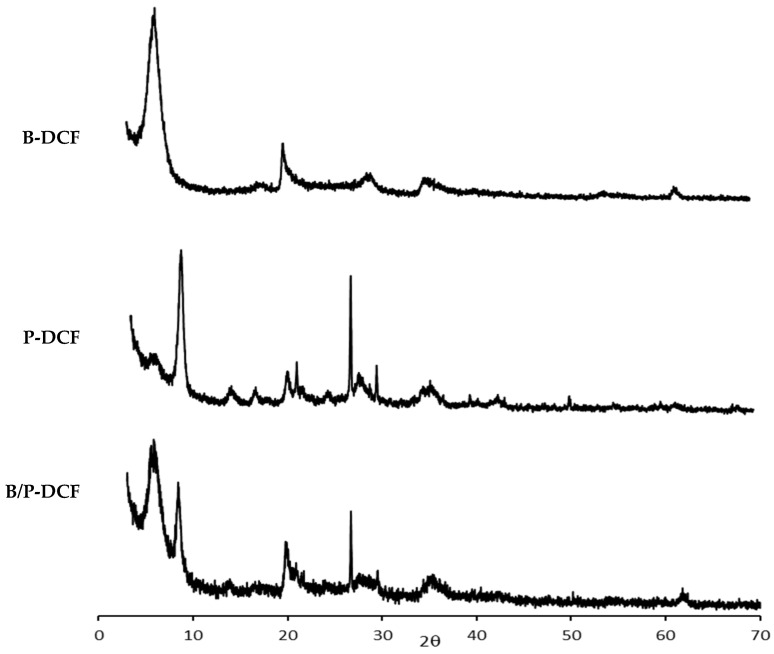
XRD of B-DCF, P-DCF, and B/P-DCF gels.

Figure 4 reports the XRD spectra of the gels (B-DCF: top panel, P-DCF: middle panel, and B/P-DCF: bottom panel). Reflection peaks of the XRD spectra are related to the presence of pure bentonite and palygorskite. In detail, B-DCF spectrum showed typical reflections of bentonite at 6° and 20° 2θ, while P-DCF hydrogel exhibited peaks at 8.5°, 21.0°, and 26.7° 2θ corresponding to the typical palygorskite pattern. Moreover, B/P-DCF hydrogel was characterized by typical features of both clays and peaks at 6 and 8.5° 2θ, and which were attributable to the presence of bentonite and palygorskite, respectively.

Although DCF nanosuspension displayed a crystalline profile, as previously reported by Pireddu et al. [19], the spectra of the gels are dominated by the presence of the clays which completely overlap the DCF nanosuspension crystalline peaks.

**Figure 5:**
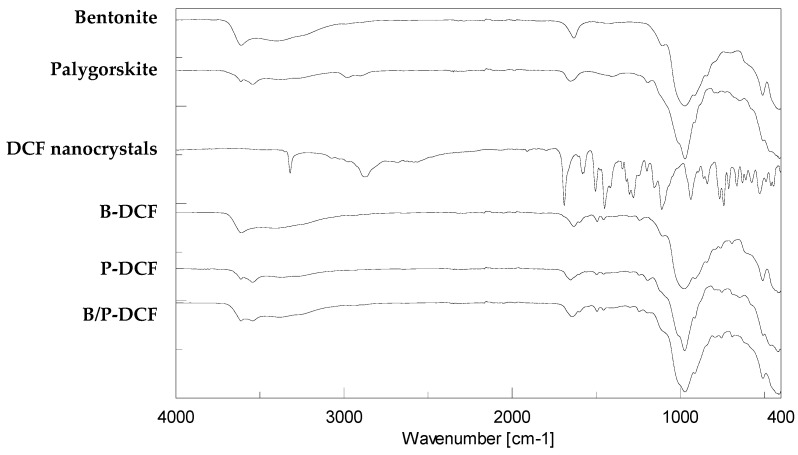
FTIR of the pristine components and gels (B-DCF, P-DCF, and B/P-DCF).

Figure 5 reports FTIR spectra of the pure components and DCF nanocrystals-loaded hydrogels.

OH groups coordinated with the octahedral cations at 3600 cm^−1^ and OH of water in the interlaminar space at 3400 cm^−1^ caused vibrations in the FTIR spectra of pure bentonite [26,27]. In addition, a strong band at 1632 cm^−1^ is related to the water molecules coupled to exchangeable cations. Si-O-Si out-of-plane and in-plane are responsible for the bands at 1100 cm^−1^ and 985 cm^−1^, respectively.

As for pristine palygorskite, OH stretching vibrations determined bands at 3616 cm^−1^ and 3542 cm^−1^ due to the Si-OH bond. The band at 1652 cm^−1^ was related to the bending of absorbed and zeolitic water, while silicate bands are noticeable in the range of 1400–1200 cm^−1^ [28].

The FTIR spectra of DCF nanosuspension was ascribable to Poloxamer and DCF, used for nanocrystals preparation. In particular, the main absorption bands are NH stretching at 3325 cm^−1^ and CH stretching at 2890 cm^−1^. The spectrum also showed distinct absorption bands due to C=O at 1691 cm^−1^, and COO-symmetric and asymmetric stretching at 1578 and 1452 cm^−1^, respectively.

The presence of the DCF in the hydrogels was difficult to confirm due to the overlapping of vibrations. Although the hydrogels’ spectra are overlapped by the presence of clay, the vibrations at 1691 cm^−1^ related to C=O stretching are attributable to the presence of DCF nanocrystals with low intensity due to the lower content of the drug in relation to the other components. Moreover, these bands were not detected in both pristine bentonite and palygorskite. In addition, no strong interaction between DCF and clay occurred since no band shifts in the FTIR spectra were detected.

**Figure 6:**
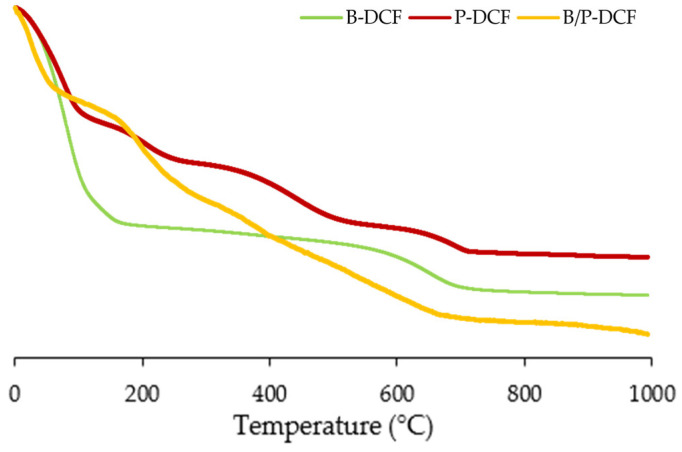

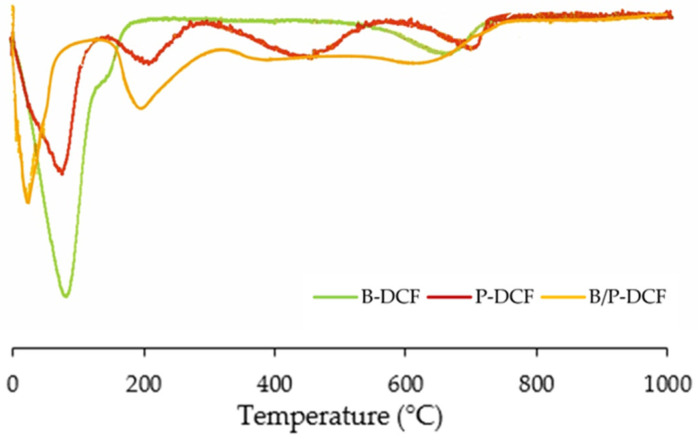
TGA (**top panel**) and DSC (**bottom panel**) of the gels (B-DCF, P-DCF, and B/P-DCF).

In Figure 6, TGA (top panel) and DSC (bottom panel) spectra of diclofenac nanocrystals-loaded inorganic hydrogels (B-DCF, P-DCF, and B/P-DCF) have been reported.

As previously reported, TGA profile of pristine bentonite is characterized by first slight weight loss due to water evaporation, followed by clay dehydroxylation in the temperature higher than 600 °C [26]. On the other hand, pure palygorskite highlighted a degradation in four stages: a first weight loss corresponding to surface water loss, a second stage at around 220 °C related to zeolitic water, a third event corresponding to the water linked to the octahedral ions, and finally, a mass loss due to the dehydroxylation at a temperature higher than 600 °C [28].

All of the hydrogels showed a first water loss followed by a two-stages degradation at a temperature higher than 200 °C. In general, independently of the composition, the hydrogels seem to protect DCF nanocrystals from thermal degradation, which occurred at a higher temperature compared to the pristine components (50 °C, due to poloxamer melting, followed by a drug degradation at around 150–152 °C) [12].

Regarding DSC analysis, the hydrogels revealed a first endotherm between 44 and 155 °C corresponding to water evaporation. In addition, endothermic events of DCF nanosuspension-loaded hydrogels occurred over 200 °C, confirming a stability effect of the systems. These results agreed with weight losses of TGA analysis, and demonstrated that the inclusion of DCF in clays-based hydrogels resulted in an increased thermal stability.

### 3.4. In Vitro Release Study of DCF Nanocrystals-Loaded Hydrogels

**Figure 7:**
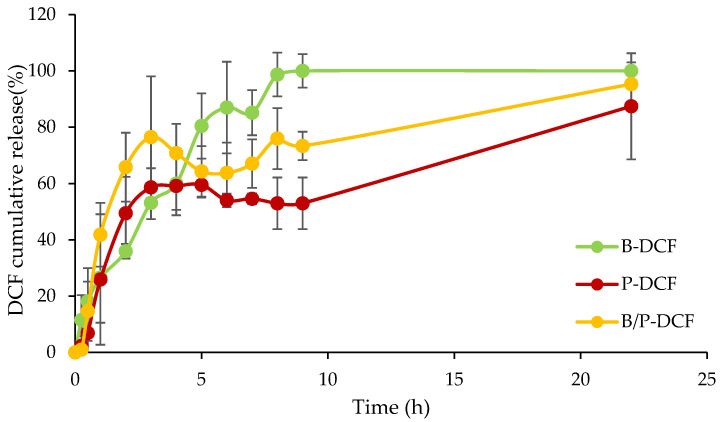
DCF cumulative release from B-DCF, P-DCF, and B/P-DCF gels (mean values ± s.d.; n = 3).

Figure 7 reports the release profiles of DCF from B-DCF, P-DCF, and B/P-DCF gels. B-DCF hydrogels were characterized by the higher profile, followed by B/P-DCF, and finally by P-DCF hydrogels. Similar release profiles were obtained for both P-DCF and B/P-DCF hydrogels, which can be divided into two stages: an initial fast release during the first 3 h, in which approximately 75% and 58% of the drug was released from P-DCF and B/P-DCF hydrogels, respectively, followed by a slow and prolonged release up to 22 h. These release profiles could be related to the systems’ hydration, swelling, and degradation. In the first stage, due to the hydration and a partial loss of structure, a rapid onset of DCF present in the surface layers occurred. The second stage is more closely related to the slower hydration of the hydrogel inner part, and the consequent diffusion of DCF across the hydrogel network.

The results revealed that the release was markedly affected by the hydrogels’ composition: in particular, the presence of palygorskite lead to a slower DCF release. This significant difference could be attributed to the influence of palygorskite, which may adsorb the drug and thus limit its release.

On the other hand, B-DCF hydrogels highlighted a constant release of DCF, reaching 100% of drug release after 8 h. The faster release of DCF, when the hydrogel is based on bentonite, could be related to the lower viscosity. This should facilitate entry of the dissolution medium into the system, thus dissolving and subsequently releasing the drug.

### 3.5. In-Vitro Skin Penetration/Permeation

**Figure 8:**
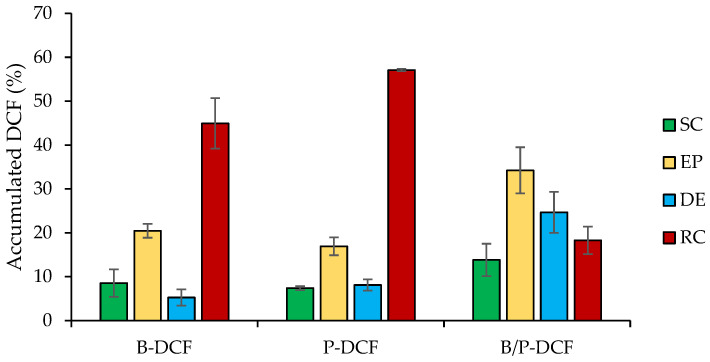
Cumulative amount (%) of DCF retained into and permeated through the skin layers (stratum corneum: SC, epidermis: EP, dermis: DE, and receptor chamber: RC) after 24 h of the application of B-DCF, P-DCF, and B/P-DCF gels (mean values ± s.d.; n = 3). ANOVA one-way; Scheffé test (*p* ≤ 0.05): EP: P-DCF vs. B/P-DCF; RC: B-DCF and P-DCF vs. B/P-DCF.

Figure 8 reports DCF skin penetration into the stratum corneum (SC), epidermis (EP), dermis (DE), and receptor chamber (RC) from B-DCF, P-DCF, and B/P-DCF hydrogels.

B-DCF and P-DCF gels caused the highest DCF amount to the deeper skin layers, while the smallest one was permeated upon the application of B/P-DCF gel. In detail, the presence of bentonite or palygorskite provided a significant increase of DCF skin permeation in the receptor chamber (2.5-fold higher than B/P-DCF).

Moreover, B/P-DCF hydrogel led to a significant increase of DCF concentration in skin epidermis. According to Liu et al. [29], bundles of elongate and lath-like particles of palygroskite would interact with the lamellas of bentonite as a result of the hydrogen-bond interaction between the hydroxyl groups. Therefore, the lower DCF accumulation of B/P-DCF hydrogel in the deeper skin layers could be attributable to a certain degree of interaction between palygorskite and bentonite, leading to a decrease in mobility of the nanocrystals.

In our previous studies [12,30], we performed an in vitro skin penetration/permeation test of DCF nanosuspensions, proving the ability of the nanocrystals to improve the dermal drug bioavailability of poorly water-soluble drugs. The application of the nanocrystals suspension on the skin is not feasible as it is a liquid formulation consisting of mostly water, which would cause leakage from the application site and reduced drug absorption. Therefore, incorporation of the nanocrystals inside the clay hydrogels is needed to overcome these issues. Owing to the higher viscosity of the clay hydrogels prepared here, compared to the liquid nanosuspension, they can be applied on the skin and left in place for longer time, thus enhancing DCF penetration to the deeper skin layers.

## 4. Conclusions

In this work, DCF nanocrystals-loaded clay-based hydrogels were successfully developed and characterized. DCF nanosuspension was manufactured using the wet ball media milling technique, and then loaded in clay-based hydrogels by means of the homogenization method. The presence of different clays affected the hydrogels morphology, and the EDX analysis confirmed the efficient incorporation of DCF into the 3D network of the hydrogels. In addition, independently of the composition, the hydrogels seem to protect DCF nanocrystals from thermal degradation, which occurred at a higher temperature compared to the pristine components. The in vitro release and skin penetration/permeation revealed that the release was markedly affected by the hydrogels’ composition: in particular, the presence of both palygorskite and bentonite seems to decrease the mobility of the nanocrystals, and consequently its release and penetration into the skin. In conclusion, bentonite- or palygorskite-based hydrogels show great potential as an alternative strategy to enhance the topical bioavailability of DCF nanocrystals.

## Figures and Tables

**Table 1:** Quali-quantitative composition and characterization of DCF nanosuspension.

DCF Nanosuspension Composition		Characterization		
Component	% (*w*/*w*)	Mean Diameter (nm)	PDI	Zeta Potential (mV)
DCF	1.0	287.8 ± 10.1	0.25 ± 0.04	−26.8 ± 0.7
Poloxamer 188	0.5			
Water	98.5			

## Data Availability

Data available on request.

## References

[1] García-Villén F., Sánchez-Espejo R., López-Galindo A., Cerezo P., Viseras C. (2020). Design and characterization of spring water hydrogels with natural inorganic excipients. Appl. Clay Sci..

[2] Dawson J.I., Kanczler J.M., Yang X.B., Attard G.S., Oreffo R.O. (2011). Clay gels for the delivery of regenerative microenvironments. Adv. Mater..

[3] Bardziński P.J. (2014). On the impact of intermolecular interactions between the quaternary ammonium ions on interlayer spacing of quat-intercalated montmorillonite: A molecular mechanics and ab-initio study. Appl. Clay Sci..

[4] Lu Y., Wang A. (2022). From structure evolution of palygorskite to functional material: A review. Micropor Mesopor Mat..

[5] Cui J., Zhang Z., Han F. (2020). Effects of pH on the gel properties of montmorillonite, palygorskite and montmorillonite-palygorskite composite clay. Appl. Clay Sci..

[6] Simonton T.C., Komarneni S., Roy R. (1988). Gelling properties of sepiolite versus montmorillonite. Appl. Clay Sci..

[7] López-Galindo A., Viseras C., Aguzzi C., Cerezo P., Galàn E., Singer A. (2011). Chapter 13—Pharmaceutical and Cosmetic Uses of Fibrous Clays. Developments in Clay Science.

[8] Aguzzi C., Viseras C., Cerezo P., Rossi S., Ferrari F., López-Galindo A., Caramella C. (2005). Influence of dispersion conditions of two pharmaceutical grade clays on their interaction with some tetracyclines. Appl. Clay Sci..

[9] Carazo E., Borrego-Sánchez A., García-Villén F., Sánchez-Espejo R., Cerezo P., Aguzzi C., Viseras C. (2018). Advanced Inorganic Nanosystems for Skin Drug Delivery. Chem. Rec..

[10] Nugrahani I., Utami D., Ibrahim S., Nugraha Y.P., Uekusa H. (2018). Zwitterionic cocrystal of diclofenac and l-proline: Structure determination, solubility, kinetics of cocrystallization, and stability study. Eur. J. Pharm. Sci..

[11] Lai F., Sinico C., Ennas G., Marongiu F., Marongiu G., Fadda A.M. (2009). Diclofenac nanosuspensions: Influence of preparation procedure and crystal form on drug dissolution behaviour. Int. J. Pharm..

[12] Pireddu R., Sinico C., Ennas G., Marongiu F., Muzzalupo R., Lai F., Fadda A.M. (2015). Novel nanosized formulations of two diclofenac acid polymorphs to improve topical bioavailability. Eur. J. Pharm. Sci..

[13] Pireddu R., Schlich M., Marceddu S., Valenti D., Pini E., Fadda A.M., Lai F., Sinico C. (2020). Nanosuspensions and Microneedles Roller as a Combined Approach to Enhance Diclofenac Topical Bioavailability. Pharmaceutics.

[14] Schlich M., Casula L., Musa A., Pireddu R., Pitzanti G., Cardia M.C., Valenti D., Marceddu S., Fadda A.M., De Luca M.A. (2022). Needle-Free Jet Injectors and Nanosuspensions: Exploring the Potential of an Unexpected Pair. Pharmaceutics.

[15] Ruggeri M., Sánchez-Espejo R., Casula L., Barbosa R.D.M., Sandri G., Cardia M.C., Lai F., Viseras C. (2022). Clay-Based Hydrogels as Drug Delivery Vehicles of Curcumin Nanocrystals for Topical Application. Pharmaceutics.

[16] Lee M.H., Shin G.H., Park H.J. (2017). Solid lipid nanoparticles loaded thermoresponsive pluronic–xanthan gum hydrogel as a transdermal delivery system. J. Appl. Polym. Sci..

[17] Nawaz A., Ullah S., Alnuwaiser M.A., Rehman F.U., Selim S., Al Jaouni S.K., Farid A. (2022). Formulation and Evaluation of Chitosan-Gelatin Thermosensitive Hydrogels Containing 5FU-Alginate Nanoparticles for Skin Delivery. Gels.

[18] Simon A., Amaro M.I., Healy A.M., Cabral L.M., de Sousa V.P. (2016). Comparative evaluation of rivastigmine permeation from a transdermal system in the Franz cell using synthetic membranes and pig ear skin with in vivo-in vitro correlation. Int. J. Pharm..

[19] Pireddu R., Caddeo C., Valenti D., Marongiu F., Scano A., Ennas G., Lai F., Fadda A.M., Sinico C. (2016). Diclofenac acid nanocrystals as an effective strategy to reduce in vivo skin inflammation by improving dermal drug bioavailability. Coll. Surf. B..

[20] Ahuja M., Dhake A.S., Sharma S.K., Majumdar D.K. (2009). Stability studies on aqueous and oily ophthalmic solutions of diclofenac. Yakugaku Zasshi.

[21] Romero G.B., Chen R., Keck C.M., Müller R.H. (2015). Industrial concentrates of dermal hesperidin smartCrystals^®^--production, characterization & long-term stability. Int. J. Pharm..

[22] Chemeda Y.C., Christidis G.E., Khan N.M.T., Koutsopoulou E., Hatzistamou V., Kelessidis V.C. (2014). Rheological properties of palygorskite–bentonite and sepiolite–bentonite mixed clay suspensions. Appl. Clay Sci..

[23] Luckham P.F., Rossi S. (1999). The colloidal and rheological properties of bentonite suspensions. Adv. Colloid. Interface Sci..

[24] Heller H., Keren R. (2001). Rheology of Na-Rich Montmorillonite Suspension as Affected by Electrolyte Concentration and Shear Rate. Clays Clay Min..

[25] Neaman A., Singer A. (2000). Rheology of Mixed Palygorskite-Montmorillonite Suspensions. Clays Clay Min..

[26] García-Villén F., Faccendini A., Aguzzi C., Cerezo P., Bonferoni M.C., Rossi S., Grisoli P., Ruggeri M., Ferrari F., Sandri G. (2019). Montmorillonite-norfloxacin nanocomposite intended for healing of infected wounds. Int. J. Nanomed..

[27] Faccendini A., Ruggeri M., Rossi S., Bonferoni M.C., Aguzzi C., Grisoli P., Viseras C., Sandri G., Ferrari F. (2020). Norfloxacin loaded electrospun scaffolds: Montmorillonite nanocomposite vs. free drug. Pharmaceutics.

[28] Carazo E., Borrego-Sánchez A., García-Villén F., Sánchez-Espejo R., Viseras C., Cerezo P., Aguzzi C. (2018). Adsorption and characterization of palygorskite-isoniazid nanohybrids. Appl. Clay Sci..

[29] Liu Y., Kang Y., Mu B., Wang A. (2014). Attapulgite/bentonite interactions for methylene blue adsorption characteristics from aqueous solution. J. Chem. Eng..

[30] Pireddu R., Sinico C., Ennas G., Schlich M., Valenti D., Murgia S., Marongiu F., Fadda A.M., Lai F. (2018). The effect of diethylene glycol monoethyl ether on skin penetration ability of diclofenac acid nanosuspensions. Colloids Surf. B Biointerfaces.

